# Structural and Functional Study of the GlnB22-Insulin Mutant Responsible for Maturity-Onset Diabetes of the Young

**DOI:** 10.1371/journal.pone.0112883

**Published:** 2014-11-25

**Authors:** Květoslava Křížková, Václav Veverka, Lenka Maletínská, Rozálie Hexnerová, Andrzej M. Brzozowski, Jiří Jiráček, Lenka Žáková

**Affiliations:** 1 Institute of Organic Chemistry and Biochemistry, Academy of Sciences of the Czech Republic, v.v.i., Flemingovo nám. 2, 166 10 Prague 6, Czech Republic; 2 York Structural Biology Laboratory, Department of Chemistry, The University of York, Heslington, York, YO10 5DD, United Kingdom; University of South Florida College of Medicine, United States of America

## Abstract

The insulin gene mutation c.137G>A (R46Q), which changes an arginine at the B22 position of the mature hormone to glutamine, causes the monogenic diabetes variant maturity-onset diabetes of the young (MODY). In MODY patients, this mutation is heterozygous, and both mutant and wild-type (WT) human insulin are produced simultaneously. However, the patients often depend on administration of exogenous insulin. In this study, we chemically synthesized the MODY mutant [GlnB22]-insulin and characterized its biological and structural properties. The chemical synthesis of this insulin analogue revealed that its folding ability is severely impaired. *In vitro* and *in vivo* tests showed that its binding affinity and biological activity are reduced (both approximately 20% that of human insulin). Comparison of the solution structure of [GlnB22]-insulin with the solution structure of native human insulin revealed that the most significant structural effect of the mutation is distortion of the B20-B23 β-turn, leading to liberation of the B chain C-terminus from the protein core. The distortion of the B20-B23 β-turn is caused by the extended conformational freedom of the GlnB22 side chain, which is no longer anchored in a hydrogen bonding network like the native ArgB22. The partially disordered [GlnB22]-insulin structure appears to be one reason for the reduced binding potency of this mutant and may also be responsible for its low folding efficiency *in vivo*. The altered orientation and flexibility of the B20-B23 β-turn may interfere with the formation of disulfide bonds in proinsulin bearing the R46Q (GlnB22) mutation. This may also have a negative effect on the WT proinsulin simultaneously biosynthesized in β-cells and therefore play a major role in the development of MODY in patients producing [GlnB22]-insulin.

## Introduction

In 2012, about 350 million people worldwide were affected by diabetes mellitus. Type 2, or non-insulin dependent, diabetes comprises more than 90% of diabetes cases. This metabolic disease is correlated with obesity, excessive caloric intake, and increasing age. In contrast, type 1 diabetes is an autoimmune disease primarily caused by dysfunction of insulin-producing Langerhans β-cells in the pancreas. Thus, type 1 diabetics are fully dependent on administration of exogenous insulin. In addition to these two main (and a few minor) types of diabetes, there is an inherited, monogenic type of diabetes caused by single-gene mutations in the insulin gene. There are two major classes of monogenic diabetes: neonatal diabetes mellitus (NDM) and maturity-onset diabetes of the young (MODY) [1], [2]. While NDM develops within 6 months after birth, the clinical diagnosis of MODY is based on a few criteria, such as a family history of diabetes, nonketotic diabetes, and onset before 25 years of age [3].

Insulin is a small, 51-amino-acid globular protein consisting of two chains, A-chain (21 amino acids) and B-chain (30 amino acids), linked by two invariant disulfide bridges (A7-B7, A20-B19). A-chain contains an additional disulfide bridge (A6-A11) [4]. Insulin is derived from an immediate precursor, proinsulin, in which A and B chains are linked by the C-peptide, a connecting peptide with variable sequences and lengths among species. The initial translation product of insulin mRNA is preproinsulin, which contains a 24-amino-acid N-terminal signal sequence attached to proinsulin, allowing the entry of this polypeptide into the endoplasmic reticulum (ER). The conversion of preproinsulin to proinsulin occurs in the rough ER, and proinsulin is then transported into the ER lumen for disulfide pairing. Successfully folded proinsulin is subsequently transported to the Golgi apparatus, where it is packaged into immature secretory granules. During formation and maturation of the granules, proinsulin is cleaved to liberate insulin and the C-peptide [5]. Misfolded proinsulin is retained in the ER lumen and destroyed, as the accumulation of misfolded prohormone in the lumen may lead to severe ER stress, followed by progressive dysfunction and death of the β-cells [6].

Characterization of insulin analogues [7]–[16], including systematic mutational analysis [17]; the occurrence of natural insulin mutants [18]–[21]; and insights into the insulin-insulin receptor (IR) complex [22]–[24] have revealed the importance and contribution of insulin regions and individual amino acids for aggregation, folding, stability, and interaction with IR. A specific mutation in insulin or its precursors may affect both the biological and physicochemical properties of the protein. The sites of naturally occurring insulin mutations determine the age of onset and clinical severity of diabetes, which may range from a mild, accidentally diagnosed hyperglycemia to seriously advanced metabolic disorder. The most deleterious insulin mutations are alterations of cysteines or other amino acids important for the interactions with IR [18], [20]; these usually lead to NDM. Mutations at less ‘important’ positions can affect the folding of (pro)hormones, their ability to aggregate and form fibrils, and the efficiency of excision of the C-peptide. In these cases, the onset of diabetes may occur anytime during childhood or adulthood with a wide spectrum of clinical consequences.

The insulin gene mutation c.137G>A (R46Q), in which arginine at position B22 is replaced by glutamine, causes MODY, and has been identified to date in one Czech family and one Norwegian family [25], [26]. MODY patients have a heterozygous mutation, and both the mutant and WT insulin are produced simultaneously. Both families carrying this gene mutation have experienced similar symptoms. All patients were diagnosed with diabetes during youth or adolescence (13–24 years), and they have been treated with insulin, oral hypoglycaemic agents, and in some cases solely with diet [25], [26].

Here, we have characterized a naturally occurring insulin analogue bearing the ArgB22→Gln mutation implicated in MODY. This is the first study of the synthesis, purification, and biological and structural characterization of a naturally occurring MODY-causing mutant. To understand the impact of this mutation on insulin structure and functionality, we prepared the [GlnB22]-insulin analogue using total chemical synthesis, followed by A and B chain combination. We also characterized its *in vitro* and *in vivo* biological activities and determined the solution NMR structure. Our results increase understanding of the structural consequences of this naturally occurring mutation and their impact on the functionality of R46Q insulin in MODY.

## Materials and Methods

### Solid-phase peptide synthesis of analogue chains

The WT A-chain and GlnB22 B-chain were synthesized by stepwise coupling of the corresponding Fmoc-amino acids on Fmoc-Asn(Trt)-Wang LL resin or Fmoc-Thr(OtBu)-Wang LL resin (Nova Biochem, San Diego, CA, USA), respectively, using an automatic solid-phase synthesizer (ABI 433A, Applied Biosystems, Foster City, CA, USA). HBTU/HOBt in DMF was used as a coupling reagent. Both fully protected peptide chains were cleaved from the resin with a mixture of TFA/H_2_O/TIS/EDT/phenol/thioanisol (90:3:1:1:2:3).

### Sulfitolysis

Crude A- or GlnB22 B-chain in reduced (SH) form was dissolved and stirred in 25 ml sulfiltolysis buffer (100 mM Tris, 250 mM Na_2_SO_3_, 80 mM Na_2_S_4_O_6_, 7 M GuaHCl, pH 8.6) for 3 h at room temperature to convert SH groups to S-sulfonates. Chains were then desalted on a Sephadex G10 (column 4 cm×85 cm) in 50 mM NH_4_HCO_3_ and purified using RP-HPLC (Waters 600; Nucleosil C18 column, 250 mm×21 mm, 5 µm).

### Disulfide bridge combination

The combination of A-chain and GlnB22 B-chain was performed as previously described [27]. Insulin A-chain (30 mg) and GlnB22 B-chain (15 mg) were dissolved in 2 and 1 ml of degassed 0.1 M Gly/NaOH buffer, pH 10.5, respectively. The exact molar concentrations of individual chains were determined by UV spectrophotometry. They were combined, and dithiothreitol (DTT, aliquoted from Pierce, article number 20291) in a minimal volume of degassed 0.1 M Gly/NaOH buffer, pH 10.5, was rapidly added to the polypeptide solution to yield a SH:SSO_3_^−^ molar ratio of 1.1. The solution was stirred for 30 min in a capped vessel at room temperature. After the reduction of SSO_3_^−^ to SH, 3 ml of aerated 0.1 M Gly/NaOH buffer, pH 10.5, were added, and the resulting solution was stirred for 48 h at 4°C in an open vessel to permit air oxidation. Glacial acetic acid (3 ml) was added to the mixture to terminate the reaction. The resulting mixture was applied to a low-pressure column (Sephadex G-50 in1 M acetic acid, 2 cm×75 cm). Fractions containing the respective analogues were purified using RP-HPLC (Waters 600; NucleosilC18 column, 250 mm×8 mm, 5 µm). The molecular weight of the resulting analogues was confirmed by high-resolution mass spectroscopy (LTQ Orbitrap XL, Thermo Fisher Scientific, Waltham, MA, USA).

### Receptor binding affinity

Receptor binding studies were performed with human IM-9 lymphocytes, which containing only the IR-A isoform. *K*_d_ values were determined according to the procedure recently described by Morcavallo *et al.* [28]. Binding data were analyzed with Excel software developed specifically for the IM-9 cell system in the laboratory of Prof. Pierre De Meyts (A. V. Groth and R. M. Shymko, Hagedorn Research Institute, Denmark, a kind gift from P. De Meyts) using a non-linear regression method and one-site fitting program, taking into account potential depletion of the free ligand. The dissociation constant of human ^125^I-insulin was set to 0.3 nM.

### Experimental animals

All experiments followed the ethical guidelines for animal experiments described in law 246/1992 of the Czech Republic and were approved by the committee for experiments with laboratory animals of the Academy of Sciences of the Czech Republic. Inbred C57BL/6 male mice (Charles River, Sulzfeld, Germany) were housed at 23°C under a daily cycle of 12 h of light and dark (light from 6:00 AM) with free access to water and a standard chow diet that contained 25% calories from protein, 9% from fat, and 66% from carbohydrate. The energy content of the diet (St-1; Mlýn Kocanda, Jesenice, Czech Republic) was 3.4 kcal/g.

### Insulin tolerance test in mice

Twelve-week-old mice (weighing 23–29 g) were randomly divided into three groups of ten mice each. Prior to the test, the mice were placed into separate cages for 3 days, with free access to water and food pellets. The food was taken away 6 h before the start of the test. Mice from the first and the second groups were injected subcutaneously with human insulin or [GlnB22]-insulin at a dose of 0.75 U/kg (calculated for average weight). One unit (U) of insulin or analogue is defined as 6 nmol of the polypeptide (∼34 µg). The compounds were dissolved in a 15∶1 mixture of saline and 0.1% acetic acid. The third group of mice was injected with saline alone. Blood glucose was measured with a glucometer (Arkray, Kyoto, Japan) in a drop of blood obtained from the tail vein before the application of human insulin, [GlnB22]-insulin, or saline to determine a basal glucose level. This was followed by glucose measurements at 10, 20, 30, 45, 60, 120 and 150 min after injection of compounds or saline. The animals were given free access to food immediately after the experiment. The test was repeated after 14 days using the same protocol. The animals were randomly divided during this process, with none receiving the same compound twice. The decrease in molar concentration of blood glucose in mmol/l (Δ c_M_) was adjusted for the impact of conditions during testing (represented by the application of saline). The data were analysed in GraphPad Prism 5.0 (San Diego, USA) and are presented as mean ±S.E.M. The significance of the changes induced by treatment was calculated using two-tailed *t*-test for independent samples.

### NMR spectroscopy

NMR spectra were acquired from a 0.35 ml sample of 0.2 mM [GlnB22]-insulin and 4 mM WT insulin in 20% d_4_-acetic acid (pH 1.9). All NMR data were collected at 25°C on a 600 MHz Bruker Avance spectrometer equipped with a triple-resonance (^15^N/^13^C/^1^H) cryoprobe. A series of homonuclear spectra were recorded to determine sequence-specific resonance assignments for both proteins: 2D TOCSY with 60 ms mixing time, 2D DQF-COSY, and 2D NOESY, which was acquired with an NOE mixing time of 200 ms. Residues involved in forming stable backbone hydrogen bonds were identified by monitoring the rate of backbone amide exchange in 2D TOCSY spectra of [GlnB22]-insulin dissolved in 20% d_4_-acetic acid/80% D_2_O. In addition, the higher concentration of WT insulin allowed for acquisition of heteronuclear ^13^C/^1^H and ^15^N/^1^H HSQC spectra and further extension of the assignments for the ^13^C and ^15^N resonances. The families of converged structures for both [GlnB22]-insulin and WT insulin were initially calculated using Cyana 2.1 [29]. A combined automated NOE assignment and structure determination protocol was used to automatically assign the NOE cross-peaks identified in the 2D NOESY spectrum and to produce preliminary structures. Backbone torsion angle constraints for WT insulin were generated from assigned chemical shifts using the program TALOS+ [30]. Subsequently, five cycles of simulated annealing combined with redundant dihedral angle constraints (Redac) [31] were used to produce sets of converged structures with no significant restraint violations (distance and van der Waals violations <0.2 Å and dihedral angle constraint violation <5°), which were further refined in explicit solvent using the YASARA software with the YASARA forcefield [32]. The structures with the lowest total energy were selected. Analysis of the family of structures obtained was carried out using the programs Molmol, iCING [33], [34], and PyMol (www.pymol.org).

## Results

### Synthesis of [GlnB22]-insulin by chain combination

We prepared the [GlnB22]-insulin analogue by total chemical synthesis and chain combination. Individual GlnB22 A- and B-chains in a 2∶1 molar excess, protected by S-sulfonate groups, were combined by oxidative recombination after reduction of S-sulfonates with a nearly stoichiometric amount of dithiothreitol. Oxidized reaction mixtures were desalted, analyzed, and purified by RP-HPLC. The molecular weight of all resulting analogues was confirmed by HR-MS. The starting amount of B chain-S-sulfonate was a limiting factor in the chain recombination reaction, and the yield of [GlnB22]-insulin was approximately 2%. In comparison, the average recombination yields of human insulin in our laboratory are in the ∼8–12% range (data not shown).

### Binding affinity of [GlnB22]-insulin to the insulin receptor

**Figure 1:**
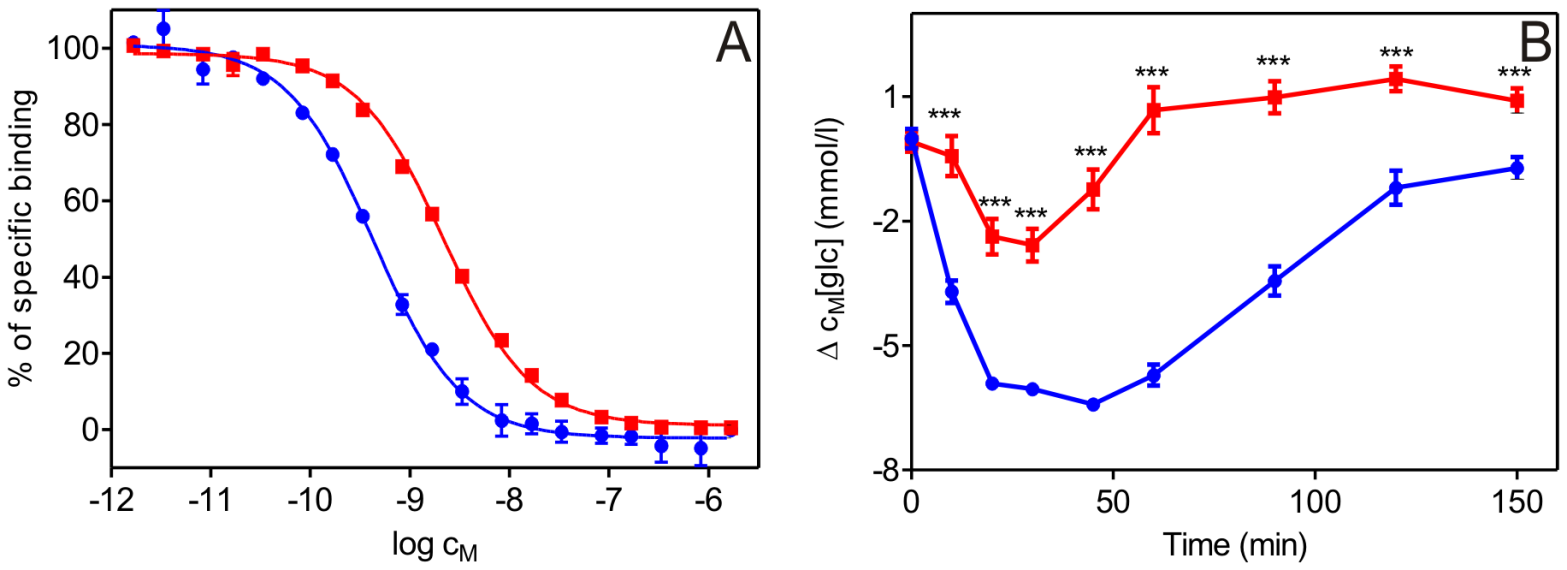
Binding affinities and *in vivo* activities of human insulin and [GlnB22]-insulin. (A) Inhibition of the binding of human [^125^I]-insulin to the plasma membrane of IM-9 cells by human insulin (blue) and [GlnB22]-insulin (red). (B) Insulin tolerance test of human insulin (blue) and [GlnB22]-insulin (red). Values are mean ±S.E., *n* = 10/group. ***p<0.01 compared to human insulin-treated group. Δ c_M_ is the decrease in molar concentration of blood glucose in mmol/l adjusted for the impact of conditions during testing (represented by the application of saline).

The binding affinity of the [GlnB22]-insulin analogue was determined using IM-9 lymphocytes, which exclusively express IR-A [35] and represent a reliable and well-established model for IR binding [36]. The binding affinity of [GlnB22]-insulin was 19.9% that of human insulin (Table 1; the corresponding binding curves are shown in Figure 1A).

**Table 1:** Values of *K*_d_ and relative binding affinities of wild-type insulin and [GlnB22]-insulin.

	*K*_d_±S.E.	Potency^a^
	nM (n)	%
Wild-type insulin	0.42±0.03 (3)	100±7
[GlnB22]-insulin	2.1±0.02 (4)	19.9±0.2

a Relative receptor binding affinity (potency) is defined as (*K_d_* of human insulin/*K_d_* of analogue) ×100.

### Insulin tolerance test in mice

[GlnB22]-insulin was administered subcutaneously to mice. [GlnB22]-insulin caused a statistically significant decrease in blood glucose levels, but its effect was shallower and shorter than that of human insulin. Nevertheless, both WT insulin and [GlnB22]-insulin have a similar time-dependent effect on blood glucose concentration (Figure 1B). The *in vivo* potency of the analogue is similar to its IR binding affinity (ca. 20% that of human insulin, Table 1).

### Solution structures of [GlnB22]-insulin and WT insulin

The overall good quality of NMR spectra suggested that both [GlnB22]-insulin and WT insulin are monomeric in 20% d_4_-acetic acid (pH 1.9). We achieved essentially complete sequence-specific assignment of ^1^H NMR resonances for both molecules using a combination of homonuclear 2D TOCSY, NOESY, and DQF-COSY experiments. The assignment of WT-human insulin was further extended to the ^15^N and ^13^C resonances using heteronuclear correlation spectra. In particular, >98% of all proton resonances were assigned in [GlnB22]-insulin and WT insulin, with the exception of the chemical exchange broadened amide proton signals from SerA9 and CysA11 in [GlnB22]-insulin, the unresolved H^ζ^ signal of PheB24, and missing H^α^ and H^β^ signals of CysA11 in both proteins. The ^1^H resonance assignments obtained for [GlnB22]-insulin and WT insulin were used for automated assignment of the NOEs identified in 2D NOESY spectra implemented in Cyana [29], yielding unique assignments for 98% of the NOE peaks observed. The 40 satisfactorily converged [GlnB22]-insulin and 50 WT insulin structures obtained from 100 random starting conformations using NMR-derived structural constraints, including distance restraints for hydrogen and disulfide bonds and additional dihedral angle restraints for WT-human insulin, were further refined in explicit water using YASARA [32]. The numbers of observed NOE peaks, distance constraints, and structural statistics for obtained structures are given in Table 2. The structures, NMR constraints, and resonance assignments for [GlnB22] and WT human insulin have been deposited in the Protein Data Bank (PDB, accession numbers 2mvd and 2mvc, respectively) and the BMRB database (accession numbers 25261 and 25260, respectively).

**Table 2:** The numbers of observed NOE peaks, additional constraints and structural statistics for calculated structures.

	*[GlnB22]-insulin*		*WT-human insulin*	
*Non-redundant distance and angle constrains*				
Total number of NOE constraints	556		859	
Short-range NOEs (i, i+1)	327		467	
Medium-range NOEs (i, i>1 i≤4)	109		201	
Long-range NOEs (i, i≥5)	117		188	
Torsion angles	-		64	
Hydrogen bond restrains	64		-	
Restricting constraints per restrained residue	12.4		18.1	
*Residual constraint violations*				
Distance violations per structure				
0.1–0.2 Å	9.2		0.34	
0.2–0.5 Å	4.95		0.02	
>0.5 Å	0		0	
r.m.s. of distance violation per constraint	0.04 Å		0.01 Å	
Maximum distance violation	0.50 Å		0.21 Å	
Dihedral angle violations per structure				
1–10°	-		1.68	
>10°	-		0	
r.m.s. of dihedral violations per constraint	-		0.70°	
Maximum dihedral angle violation	-		5.10°	
*Ramachandran plot summary from Procheck*				
Most favoured regions	97.6 %		92.4 %	
Additionally allowed regions	2.1 %		7.6 %	
Generously allowed regions	0.3 %		0.0 %	
Disallowed regions	0.0 %		0.0 %	
*r.m.s.d. to the mean structure*	ordered	all residues	ordered	all residues
All backbone atoms	0.7 Å	2.0 Å	0.6 Å	0.7 Å
All heavy atoms	1.2 Å	2.5 Å	1.0 Å	1.1 Å

**Figure 2:**
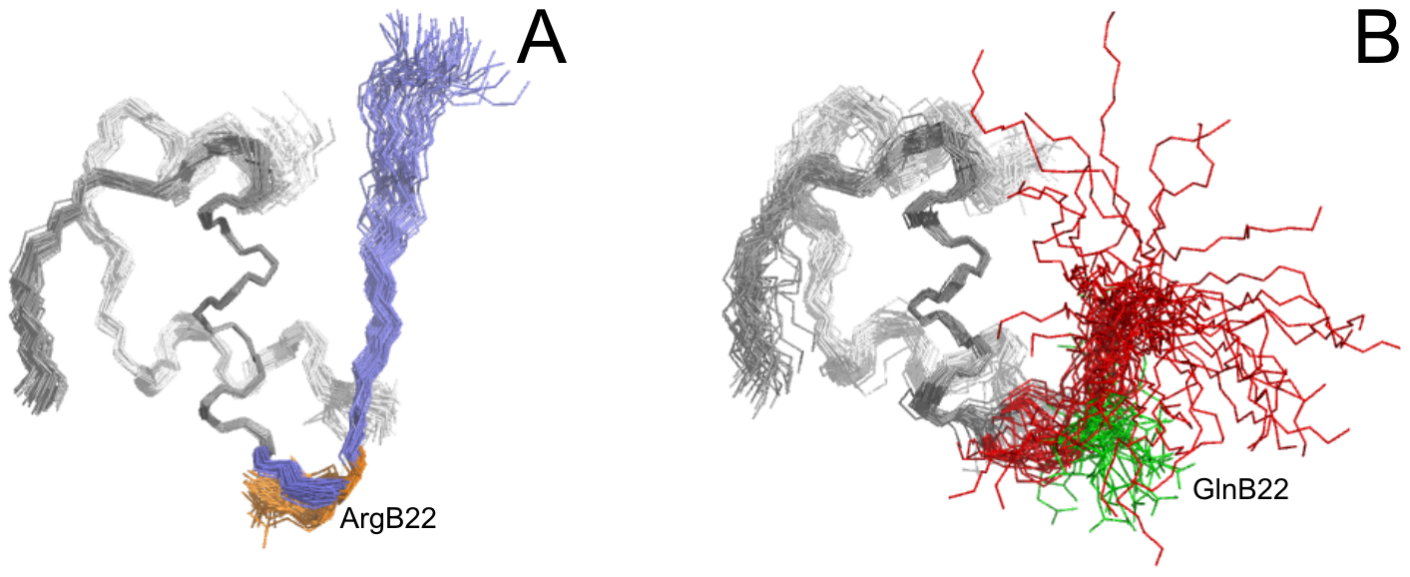
NMR structures of WT human insulin and [GlnB22]-insulin. (A) Structures of WT human insulin are represented by a best-fit superposition of the protein backbone for 50 converged structures, with the positions of arginine B22 side chains colored in orange. B20–B30 protein backbone in WT human insulin is colored in blue. (B) Structures of [GlnB22]-insulin are represented by a best-fit superposition of the protein backbone for 40 converged structures, with the positions of glutamine B22 side chains colored in green. B20–B30 protein backbone in [GlnB22]-insulin is colored in red. A1–A21 and B1-B19 protein backbones in both insulins are colored in grey.

**Figure 3:**
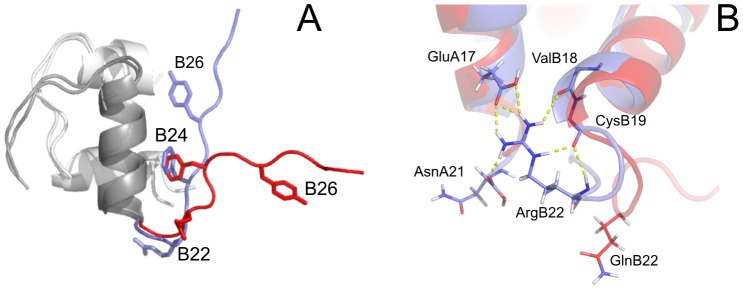
Overlay of representative NMR structures of WT human insulin and [GlnB22]-insulin. (A) Overlay of the representative structures of WT human insulin (blue) and [GlnB22]-insulin (red). (B) Detailed view of an overlay of the B20–B23 β-turn and its surrounding area in the representative structures of WT human insulin (blue) and [GlnB22]-insulin (red). The network of hydrogen bonds stabilizing the B20–B23 β-turn in WT human insulin is highlighted by dashed yellow lines.

The structures of both WT and [GlnB22]-insulin (Figures 2A and 2B, respectively, and overlay of both in Figure 3A) revealed that α-helices of the A chain and the central α-helix of the B chain are unaffected by the mutation. The N-terminus of the B-chain in both structures adopts the T-state conformation typical of solution insulin structures.

Our structure of WT human insulin is well-defined (Figures 2A and 3A); it preserves all typical structural features present in a crystalline T6 hexamer [4] and is highly similar to the previously published NMR structure of human insulin determined under acidic conditions [37]. However, there are minor differences between the structures in the close contacts between the N-terminus of the A-chain (IleA2 and ValA3) and the C-terminus of the B-chain (TyrB26, ThrB27, ProB28), where we identified no substantial NOE crosspeaks between protons in the 2D NOESY spectrum. More importantly, the family of 50 converged structures of WT insulin, accomplished by a simulated annealing calculation in explicit water using NMR-derived constraints, allowed for a more representative view of the polar contacts established between the positively charged side chain of ArgB22 and the rest of the molecule in solution. ArgB22 participates in a network of hydrogen bonds that includes the main chain of ValB18 and CysB19 and the side chains of GluA17 and AsnA21, which contribute to the stabilization of the B20–B23 β-turn in a well-ordered conformation, as illustrated in Figure 3B.

The most important structural features observed in [GlnB22]-insulin are the distortion of the B20–B23 β-turn and altered orientation of the C-terminal part of the B-chain in comparison with WT insulin (Figure 3A). The extended conformational freedom of the GlnB22 side chain (Figure 2B), which is no longer anchored in a network of hydrogen bonds like the native ArgB22, leads to the disappearance of a stabilizing B20CO-B23NH hydrogen bond (Figure 3B), and the subsequent liberation of the C-terminus from the protein core. This likely decreases the stability of the B20–B23 β-turn, which is shifted from its original position in WT insulin by ∼4-5 Å (the distance between the Cα atoms of GlnB22 and ArgB22). The destabilization of the B20–B23 β-turn subsequently affects the B24–B30 region of [GlnB22]-insulin, the conformation of which is different than in other analogues with partial detachment of the B24–B30 chain [7], [23]. Although the B21–B30 backbone of [GlnB22]-insulin departs from the WT-like direction by an angle of ∼75°, PheB24 still occupies its hydrophobic pocket, although it approaches the cavity from a different direction (Figure 3A). The amino acids B25–B30 are disordered; the movement of ThrB30Cα within all 40 structures covers a range of ∼20 Å.

## Discussion

In this study, we investigated the biological and structural relationship of the naturally occurring insulin mutant [GlnB22]-insulin, which is responsible for development of MODY.

We found that [GlnB22]-insulin has reduced binding affinity (approximately 20% of WT) for IR, which corresponds to its lower and shorter *in vivo* effect in an insulin tolerance test in mice (Table 1, Figure 1). The biological potency of this mutant matches the biological potencies of other known B22-modified analogues. Interestingly, although ArgB22 is evolutionarily conserved (except in guinea pig and porcupine insulins, which have an Asp in the B22 position [38], [39]), the B22 site seems to be tolerant to a wide range of substitutions without detrimental effects on biological potency. For example, insulins in which the positively charged B22 arginine is replaced with aspartic or glutamic acid retain about 50% of the WT biological potency [40], [41], similar to the LysB22 mutant [42]. Even the introduction of a structurally constraining α-aminoisobutyric acid (Aib) at B22 results in 8% of WT binding affinity [43], whereas incorporation of Aib at other structurally important regions of insulin have negative effects on its IR binding affinity [27], [44]. Studies with despentapeptide(B25–B30)-insulins (DPIs) revealed reduced, but not damaging, biological activities of various B22-substituted DPI analogues [45]. The only exception among all B22-substituted insulin analogues is the [AlaB22]-insulin mutant, which has 4-fold higher IR binding affinity (405%) than WT insulin [17]. The high affinity of this mutant is puzzling, especially in the context of a 4-fold reduction in the biological potency of [AlaB22]-DPI compared with [ArgB22]-DPI [45]. In general, the binding affinities for most B22 insulin mutants indicate that ArgB22 is not crucial for the biological activity of this hormone.

Therefore, a central issue is whether the 20% binding affinity (and similar biological potency) of [GlnB22]-insulin is sufficient for efficient control of glucose homeostasis *in vivo*. Some answer results from biological responses to clinically administered insulin degludec [46], which has comparable (or even lower) affinity for IR as [GlnB22]-insulin [47]. Other studies also show a divergence between *in vitro* and *in vivo* potency. Some analogues with IR binding affinity higher than that of human insulin show lower or equipotent *in vivo* biological activity. There also are examples of analogues with IR binding affinity lower than that of human insulin but with equipotent *in vivo* effects [9], [48], [49]. Explanations for this phenomenon could lie in different internalization or higher blood concentration of the analogues, which compensates for their lower affinity. Based on these findings, we expect that effective biosynthesis of the [GlnB22]-insulin mutant by pancreatic β-cells would preserve normal glucose homeostasis, especially because patients with this heterozygous mutation also produce endogenous WT insulin. Perhaps the ‘troublemaker’ role of [GlnB22]-insulin during development of MODY does not result primarily from its limited biological potency.

Some explanation for [GlnB22]-insulin's role in MODY may be found in the reduced folding properties of this form of the hormone. Its folding yield during chain-combination reactions was 5-fold lower than that of WT insulin, and even less in comparison with other insulin analogues prepared in our laboratory [27]. Similar decreased yields during preparation of recombinant or synthetic B22-modified analogues have been reported previously [17], [43], [50]. Furthermore, Liu *et al*. [50] showed that misfolding of a R46Q proinsulin mutant (i.e., ArgB22→GlnB22) blocked secretion of the co-expressed WT proinsulin. This resulted in accumulation of misfolded prohormones in the ER, induction of ER stress, ER stress-mediated progressive death of pancreatic β-cells, and finally the onset of MODY. Thus, it is probable that the development of MODY in [GlnB22]-insulin-producing patients has two different, but related and overlapping, causes. First, the amount of properly folded [GlnB22]-insulin produced in the β-cells will be low. Second, this misfolded mutant may have an unfavorable effect on the simultaneously biosynthesized WT insulin, leading to the cellular consequences described by Liu *et al.* [50]. Altogether, it seems that the most important implication of the mutation of arginine to glutamine at the B22 site is the mutant's impaired folding ability due to a loss of typical intra-protein interactions during folding. It has not yet been determined whether [GlnB22]-insulin is secreted into the circulation in patients with MODY. However, if it does, its concentration would be much lower than that of WT insulin in healthy subjects, and thus [GlnB22]-insulin insufficiency will be potentiated further by its lowered biological activity.

We set out to investigate the structural origins of the impaired binding affinity and foldability of [GlnB22]-insulin. In the crystal structure of WT insulin, arginine B22 is part of the B20-B23 β-turn; its side chain resides on the surface of the molecule and participates in a network of non-covalent interactions with AsnA21, CysB19, ValB18, and GluA17 [4]. To see whether these features are conserved in [GlnB22]-insulin, we attempted to solve the structure of the mutant. The analogue resisted crystallization efforts, so we determined its solution structure by NMR spectroscopy. We also determined the NMR structure of WT insulin under acidic conditions for direct comparison. The published set of WT human insulin NMR structures is limited to ten conformers [37], and our efforts resulted in a more comprehensive set of converged structures, which allowed a more accurate explanation of the [GlnB22]-insulin structure-activity relationship.

As expected, our acidic NMR solution structure of WT insulin is generally well-ordered and similar to the structure of insulin monomer known from T6 hexamers [4]. Main chain NHArgB22-COCysB19 and side chain NH1ArgB22-OEGluA17 hydrogen bonds are maintained in our solution structure of WT insulin (Figure 3B). In contrast, these interactions are not present in our [GlnB22]-insulin structure (Figure 3B). Compared with ArgB22 in WT insulin, GlnB22 in the mutant has greater conformational freedom and disorder. The loss of crucial hydrogen bonding interactions, especially with the C-terminus of the A chain, strongly affects the B chain arrangement, causing distortion of the B20–B23 β-turn in the [GlnB22]-insulin mutant. Reorganization of the B20–B23 β-turn affects the conformation of the B24–B30 terminus, which points away from the core of insulin and is rather disordered, which may help explain the negative outcome of our crystallization experiments. However, although the position of the B25–B30 backbone, and to some extent of PheB24 Cα, is very different from the position of their equivalents in the WT insulin B-chain, the side chain of PheB24 remains in the B24 hydrophobic pocket (Figure 3A). This confirms our recent finding regarding the intrinsic structural integrity of the PheB24 site [11].

Moreover, results of from a computational alanine-scanning study of various insulin monomers revealing the crucial importance of ArgB22 for proper stability of the insulin molecule are in agreement with our solution structure of [GlnB22]-insulin [51]. Molecular dynamics (MD) simulations of [AlaB22]-insulin also showed destabilization of the B20–B23 turn and of the C-terminal part of the B chain.

Therefore, the altered and partially disordered structure of the [GlnB22]-insulin B-chain C-terminus appears to be the most likely reason for the low foldability of this mutant. The structure and orientation of the B20–B23 β-turn may play an important role in this process, potentially tampering with the formation of the correct disulfide bonds in R46Q (GlnB22) proinsulin in the ER. This hypothesis is supported by the enhanced thermodynamic stability and conserved binding affinity of the structurally locked B20 and B23 D-Ala-substituted analogues, which possess a stable, native-like orientation of the B20–B23 turn [24].

However, it is difficult to provide an unambiguous explanation for the [GlnB22]-insulin mutant's lower IR binding affinity. Its overall 3-D structure does not offer an obvious answer. For example, the structure of highly active D-HisB24-insulin shows some similar structural features: enhanced flexibility, partial detachment of the C-terminal β-strand, and conserved position of PheB24. Therefore, it appears that one of the most probable reasons for the lower binding affinity of GlnB22-insulin lies in the destabilization and distortion of the B20–B23 β-turn. Although direct contacts between the ArgB22 side chain and the L1 domain of IR are not expected [23], [24], the possibility that ArgB22 may be involved in some important IR contacts (e.g., IR Site 2) that cannot be fulfilled by GlnB22 cannot be excluded. However, this possibility is contradicted by the high binding affinity (405% of WT) of AlaB22-insulin [17]. As MD simulations of this analogue indicated the destabilization of its B20–B23 turn [51], the argument for a detrimental effect of ‘high-entropy’ of the B20–30 chain on hormone: IR association is doubtful. An experimentally determined 3D structure of [AlaB22]-insulin may shed some light on this paradox.

Our NMR measurements indicated that [GlnB22]-insulin exists as a monomer in solution. This may result from the distortion of the B20–B23 β-turn and the altered direction of the C-terminus of the B-chain [52]. However, the effect of 20% acetic acid [11] cannot be excluded, as demonstrated by the monomeric behavior of WT human insulin (this study and [37]) or insulin analogues [11], [53] under these conditions. However, the weak self-association properties of B22-analogues have been observed in monomeric guinea pig and porcupine insulins (with AspB22) [38], [39] and in synthetic [GluB22]-des-B30 insulin [40]. It is possible that monomeric behavior of [GlnB22]-insulin during expression, processing, and storage may play a negative role in its correct function in the cells. The inability of this mutant to associate into dimers and hexamers may also affect processing of R46Q proinsulin to [GlnB22]-insulin. Although crystallization screening cannot be considered a reliable insight into protein conformation, it is worth noting that [GlnB22]-insulin did not form any dimeric/hexameric crystals under all known dimer/hexamer crystallization conditions.

In summary, the present study provides some insight into the structural and functional aspects of the Arg→Gln swap at the B22 position of insulin, a naturally occurring mutation (R46Q) that causes MODY. Chemical synthesis of [GlnB22]-insulin revealed its impaired folding ability, and *in vitro* and *in vivo* tests showed its reduced binding affinity and biological activity. The solution structure of this analogue shed some light onto the impact of GlnB22-induced structural distortion of the hormone molecule on its behavior *in vivo*. However, it also further underlined the molecular and biological complexity of insulin, in which a multifaceted role of each side chain presents a formidable obstacle to a clear delineation of its structural and functional purpose.
